# A new butterfly species from south Russia revealed through chromosomal and molecular analysis of the Polyommatus (Agrodiaetus) damonides complex (Lepidoptera, Lycaenidae)

**DOI:** 10.3897/CompCytogen.v11i4.20072

**Published:** 2017-11-24

**Authors:** Vladimir A. Lukhtanov, Alexander V. Dantchenko

**Affiliations:** 1 Department of Karyosystematics, Zoological Institute of the Russian Academy of Sciences, Universitetskaya nab. 1, St. Petersburg 199034, Russia; 2 Department of Entomology, St. Petersburg State University, Universitetskaya nab 7/9, St. Petersburg 199034, Russia; 3 Faculty of Chemistry, Lomonosov Moscow State University, GSP-1, Leninskiye Gory 1/13, Moscow119991, Russia

**Keywords:** Ancestral polymorphism, biodiversity, chromosomes, chromosomal fusion/fission, *COI*, cryptic species, DNA barcoding, incomplete lineage sorting, inverted meiosis, karyosystematics, molecular phylogenetics, mitochondrial introgression, phylogeography, speciation

## Abstract

Finding a new species is a rare event in easy-to-see and well-studied organisms like butterflies, especially if they inhabit well-explored areas such as the Western Palaearctic. However, even in this region, gaps in taxonomic knowledge still exist and here we report such a discovery. Using a combined analysis of chromosomal and molecular markers we demonstrate that *Polyommatus* blue populations from Daghestan (South Russia), previously identified as *P. aserbeidschanus*, represent in fact a new species which is described here as *P. australorossicus* **sp. n.** We also show that the enigmatic *Polyommatus damonides* described as a form of *Polyommatus damone* and later considered as an entity similar to *P. poseidon* or *P. ninae* is conspecific with a taxon previously known as *P. elbursicus*. As a result of our study, we propose several taxonomic changes within the *P. damonides* species complex and suggest the following new combinations: *P. damonides elbursicus* Forster, 1956, **comb. n.** and *P. damonides gilanensis* Eckweiler, 2002, **comb. n.**

## Introduction

Agrodiaetus Hübner, 1822, a subgenus of the species-rich Palaearctic genus *Polyommatus* Latreille, 1804 (Talavera et al. 2013), includes numerous species, subspecies and forms with uncertain taxonomic positions (de Lesse 1960a, b, Eckweiler and Häuser 1997, Häuser and Eckweiler 1997, Olivier et al. 1999, Carbonell 2000, 2001, Dantchenko 2000a, Przybyłowicz 2000, ten Hagen and Eckweiler 2001, Skala 2001, Lukhtanov and Dantchenko 2002a, b, Kandul et al. 2004, Wiemers 2003, Schurian and ten Hagen 2003, Vila et al. 2010, Talavera et al. 2013, Eckweiler and Bozano 2016). It was estimated to have originated only about 3 million years ago (Kandul et al. 2004) and radiated rapidly in the Western Palaearctic (Kandul et al. 2007). The last published review of the subgenus includes 120 valid species (Eckweiler and Bozano 2016). Many of them have extremely local ‘dot-like’ distributions that are restricted to particular mountain valleys in the Balkan Peninsula, Asia Minor, Transcaucasus, Iran and Central Asia (Vila et al. 2010, Lukhtanov et al. 2015a,b, Eckweiler and Bozano 2016, Vishnevskaya et al. 2016). This subgenus is a model system in studies of speciation (Lukhtanov et al. 2005, Wiemers et al. 2009), intraspecific differentiation (Dincă et al. 2013, Przybyłowicz et al. 2014), and rapid karyotype evolution (Lukhtanov and Dantchenko 2002a, Kandul et al. 2007, Vershinina and Lukhtanov 2013, 2017).

Species identification in *Agrodiaetus* is complicated. The morphology of male genitalia is uniform for most of the species. With a few exceptions, it can help to separate groups of species (Coutsis 1986), but not individual species. The differences in wing pattern and coloration (Eckweiler and Bozano 2016) as well as in the number of antennal segments (Carbonell 1993) are very subtle or nearly lacking between many *Agrodiaetus* species. The specific pubescence of costal area of forewings may be a useful morphological character to separate species in syntopy (Dantchenko and Churkin 2003), but it works only in certain cases. In spite of morphological similarity, the taxonomic and identification problems within the subgenus Agrodiaetus can be solved if chromosomal (de Lesse 1960a,b, Lukhtanov 1989) or molecular markers (Wiemers 2003, Kandul et al. 2004, 2007, Lukhtanov et al. 2005, Stradomsky and Fomina 2013), or their combination (Lukhtanov et al. 2006, 2008, 2014, 2015a,b, Vila et al. 2010, Lukhtanov and Tikhonov 2015, Shapoval and Lukhtanov 2015a, b) are applied. An unusual diversity of karyotypes is the most remarkable characteristic of *Agrodiaetus*. Species of this subgenus exhibit one of the highest ranges in chromosome numbers in the animal kingdom (Lukhtanov 2015). Haploid chromosome numbers (n) in *Agrodiaetus* range from n=10 in P. (A.) caeruleus (Staudinger, 1871) to n=134 in P. (A.) shahrami (Skala, 2001) (Lukhtanov and Dantchenko 2002a, Lukhtanov et al. 2005). Additionally, this subgenus demonstrates a high level of karyotypic differentiation with respect to chromosome size (Lukhtanov and Dantchenko 2002b) and variation in number of chromosomes bearing ribosomal DNA clusters (Vershinina et al. 2015). These differences provide reliable characters for species delimitation, description and identification (de Lesse 1960a, b, Lukhtanov and Dantchenko 2002a, b).

Here we use a combination of molecular mitochondrial (*COI*) and nuclear chromosomal (karyotype) markers to analyze the taxa and populations close to *Polyommatus damonides* (= lineage VIII in Kandul et al. 2004). This group includes the following species: *P. ninae* (Forster, 1956), *P. aserbeidschanus* (Forster, 1956), *P. australorossicus* sp. n., *P. damonides* (Staudinger, 1899), *P. lukhtanovi* (Dantchenko, 2005), *P. zarathustra* Eckweiler, 1997, *P. arasbarani* (Carbonell & Naderi, 2000) and *P. pierceae* (Lukhtanov & Dantchenko, 2002). Here we do not analyze the distantly related taxa *P. paulae* Wiemers & De Prins J., 2004, *P. huberti* (Carbonell, 1993), *P. turcicolus* (Koçak, 1977), *P. zapvadi* (Carbonell, 1993), *P. avajicus* (Blom, 1979) and *P. zardensis* Schurian & ten Hagen, 2001 which will be considered in later publications. The taxa of the *P. damonides* species complex were revised by Forster (1956, 1960, 1961), de Lesse (1963), Lukhtanov (1989), Carbonell (1993), Hesselbarth et al. (1995), Carbonell and Naderi (2000), Dantchenko (2000b, 2005) and Eckweiler and Bozano (2016). However, the species-level boundaries remain poorly defined in this complex.

## Material and methods

### Samples

**Figure 1.**
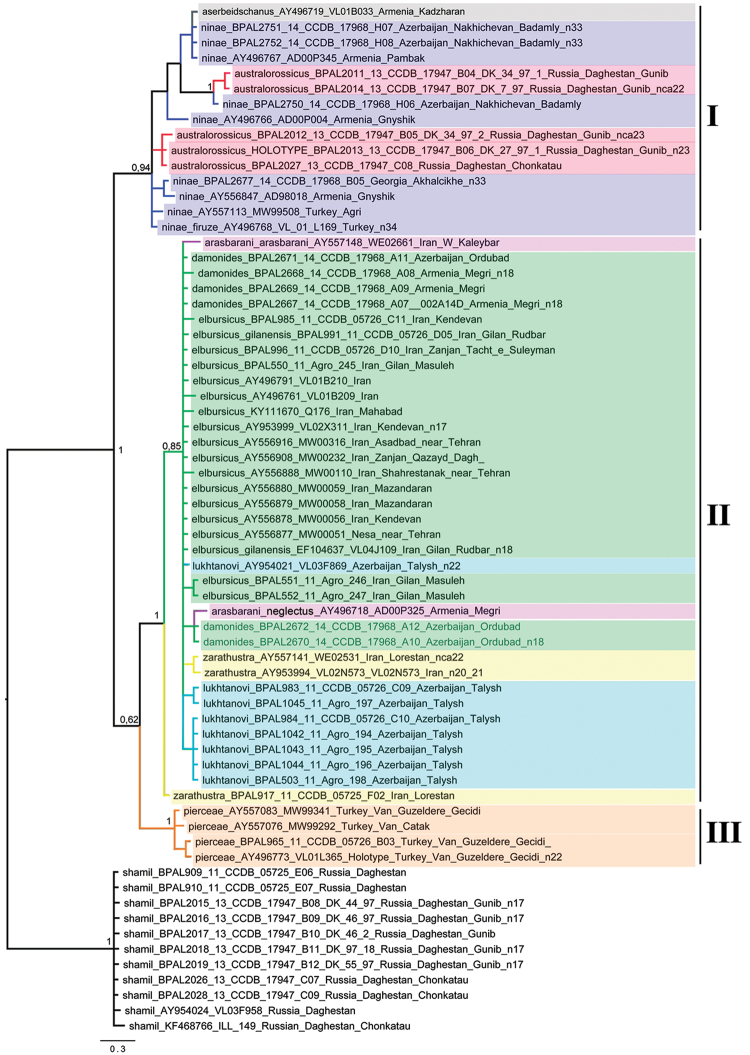
The Bayesian tree of studied *Polyommatus* samples based on analysis of the *cytochrome oxidase subunit I* (*COI*) gene. Numbers at nodes indicate Bayesian posterior probability. I, II and III are recovered haplogroups of the *P. damonides* species complex. *Polyommatus shamil*, phenotypically similar to *P. australorossicus*, but genetically distant, was used to root the tree.

Specimens examined (Supplementary Table 1, Fig. 1) are deposited in the Zoological Institute of the Russian Academy of Sciences, St. Petersburg, Russia and in the McGuire Center for Lepidoptera and Biodiversity (MGCL), Florida Museum of Natural History, University of Florida, Gainesville, Florida, USA.

### Chromosomal analysis

Karyotypes were obtained from 157 adult males representing eight species and were processed as previously described (Lukhtanov et al. 2014, 2015a, Vishnevskaya et al. 2016). Briefly, gonads were removed from the abdomen and placed into freshly prepared fixative (3:1; 96% ethanol and glacial acetic acid) directly after capturing the butterfly in the field. Testes were stored in the fixative for 3-36 months at +4°C. Then the gonads were stained in 2% acetic orcein for 30-60 days at +18-20°C. Different stages of male meiosis, including metaphase I (MI) and metaphase II (MII) were examined using an original two-phase method of chromosome analysis (Lukhtanov and Dantchenko 2002, Lukhtanov et al. 2006). In some cases diploid chromosome numbers (2n) were counted in atypical meiosis (see Lorković 1990 for a review of atypical meiosis in Lepidoptera). Abbreviation *ca* (circa) means that the count was made with an approximation due to an insufficient quality of preparation or overlapping of some chromosomes or bivalents.

### Molecular methods and DNA barcode-based phylogeographic study

Standard *COI* barcodes (658-bp 5’ segment of mitochondrial cytochrome oxidase subunit I) were studied. *COI* sequences were obtained from 30 specimens representing the *P. damonides* species group and from 9 samples of *P. shamil* (Dantchenko, 2000) which was selected as outgroup. Legs were sampled from these specimens, and sequence data from the DNA barcode region of *COI* were obtained at the Canadian Centre for DNA Barcoding (CCDB, Biodiversity Institute of Ontario, University of Guelph) using protocols described in Hajibabaei et al. (2005), Ivanova et al. (2006) and deWaard et al. (2008). Photographs of these specimens, as well as collecting data are available in the Barcode of Life Data System (BOLD), project Butterflies of Palearctic (BPAL) at http://www.boldsystems.org/. Field codes and collecting data of these samples are also shown in Figure 1.

We also used 28 published *COI* sequences (Wiemers 2003, Kandul et al. 2004, Lukhtanov et al. 2005, Kandul et al. 2007, Wiemers and Fiedler 2007, Shapoval and Lukhtanov 2016) which were downloaded from GenBank. Their accession numbers are shown in Figure 1.

The barcode analysis involved 67 *COI* sequences. Sequences were aligned using the BioEdit software (Hall 1999) and edited manually. Phylogenetic hypotheses were inferred using Bayesian inference as described previously (Vershinina and Lukhtanov 2010, Lukhtanov et al. 2016a, b). Briefly, the Bayesian analysis was performed using the program MrBayes 3.2 (Ronquist et al. 2012) with default settings as suggested by Mesquite (Maddison and Maddison 2015): burn-in=0.25, nst=6 (GTR + I + G). Two runs of 10,000,000 generations with four chains (one cold and three heated) were performed. The consensus of the obtained trees was visualised using FigTree 1.3.1 (http://tree.bio.ed.ac.uk/software/figtree/).

## Results

### Karyotypes

**Figure 2.**
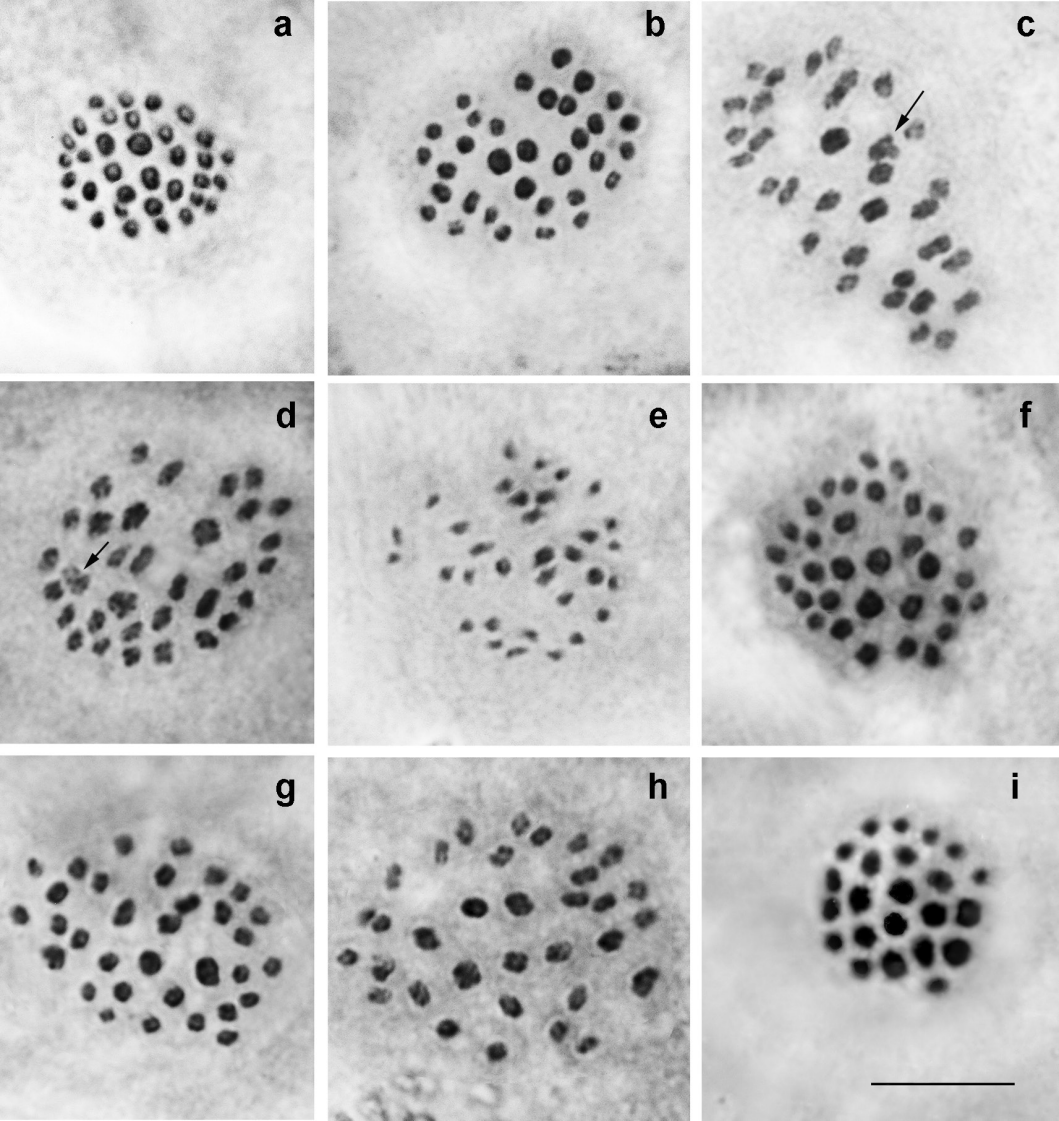
Karyotypes of *P. ninae*, *P. aserbeidschanus* and *P. australorossicus* sp. n. Trivalents are indicated by arrows. **a** *P. ninae*, sample 2014VL34, MI, n=34 **b** *P. ninae*, sample 2014VL39, MI, n=33 **c** *P. ninae*, sample 2014VL33, MI, n=32 bivalents + 1 trivalent (heterozygote for fusion/fission) **d** *P. ninae*, sample 2014VL60, n=32 bivalents + 1 trivalent (heterozygote for fusion/fission) **e** *P. ninae*, sample 2014VL33, MII, n=33 **f** *P. aserbeidschanus*, sample 05A406, MI, n=32 **g** *P. aserbeidschanus*, sample 05A387, MI, n=33 **h** *P. aserbeidschanus*, sample 05A387, MI, n=33 **i** *P. australorossicus* sp. n., sample DK-27-97-1, MI, n=23. Bar = 10 μ.

**Figure 3.**
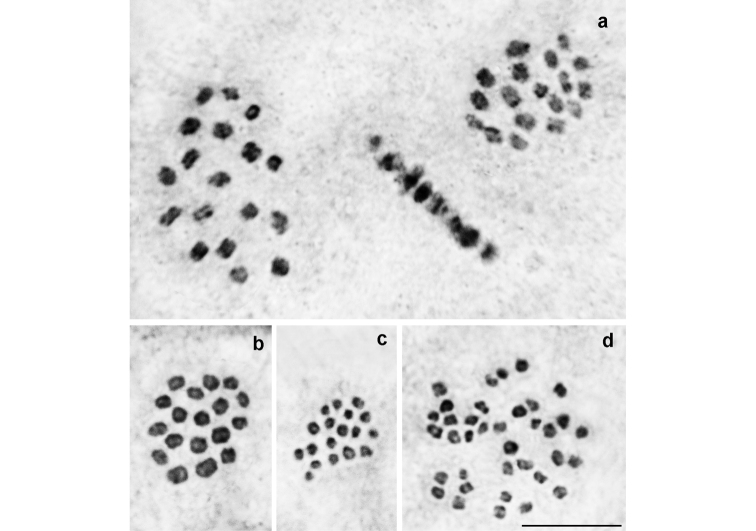
Karyotype of *P. damonides* from Ordubad vicinity in Nakhchivan (Azerbaijan) and Meghri vicinity in Armenia. **a** sample 005A14K, three MI plates (two from polar view and one from equatorial view) displaying n=18 **b** sample 005A14K, MI, n=18 **c** sample 005A14K, MII, n=18 **d** sample 2014VL04, male atypical meiosis, 2n=36. Bar = 10 μ.

**Figure 4.**
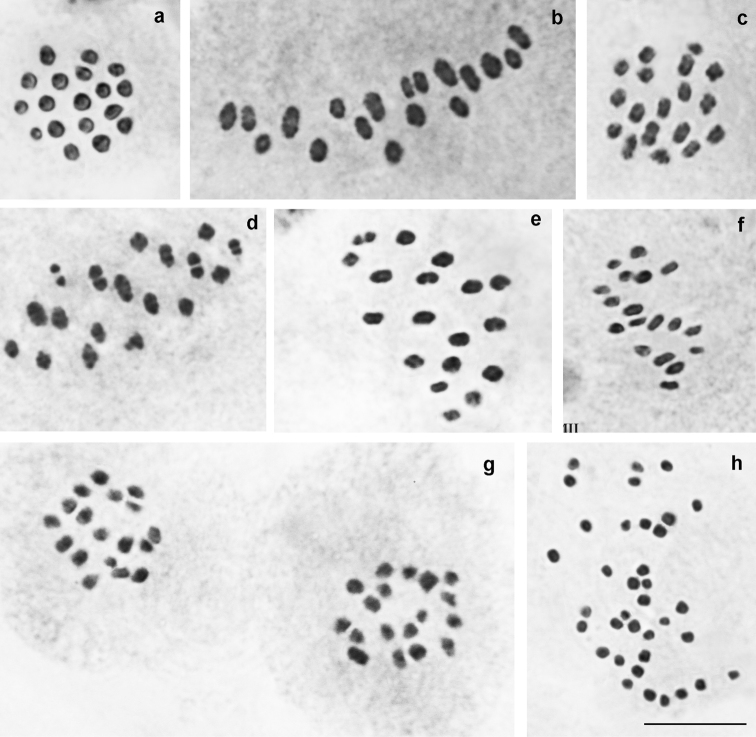
Karyotype of *P. damonides* from Iran (previously known as *P. elbursicus*). **a** sample E234, MI, n=18 **b** sample E193, MI, n=18 **c** sample E460, MI, n=18 **d** sample E237, MI, n=18 **e** sample E459, MI, n=18 **f** sample E193, MII, n=18 **g** sample J573, two sister MII plates, n=18 **h** sample E234, male atypical meiosis, 2n=36. Bar = 10 μ.

**Figure 5.**
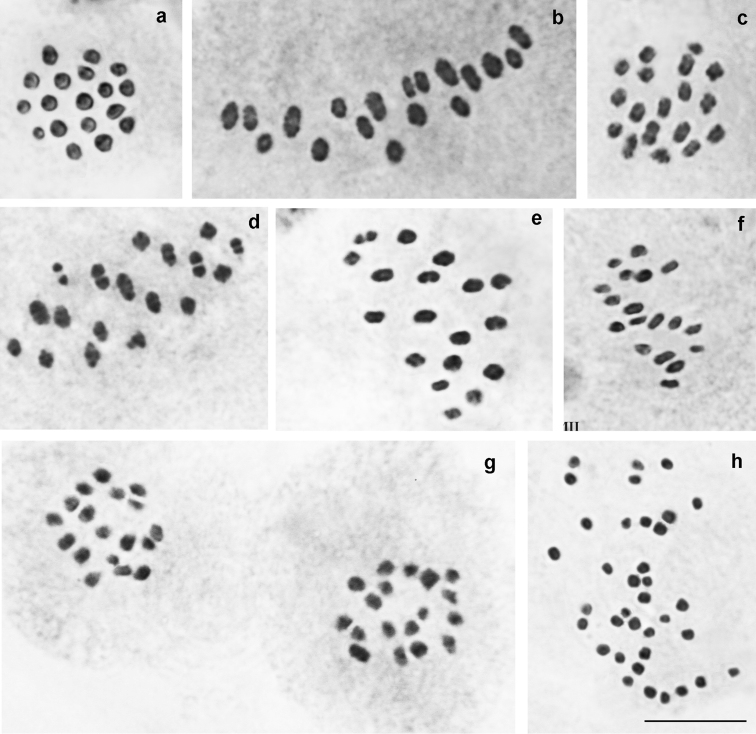
Karyotype of *P. damonides elbursicus* from Elburs Mts (north Iran). **a** sample M804, MI, n=17 **b** sample VL302, two MI plates, each displaying n=17 **c** sample VL302, diakinesis – early prometaphase, n=17 **d** sample VL302, prometaphase, n=17 **e** sample VL302, MII, n=17. Bar = 10 μ.

**Figure 6.**
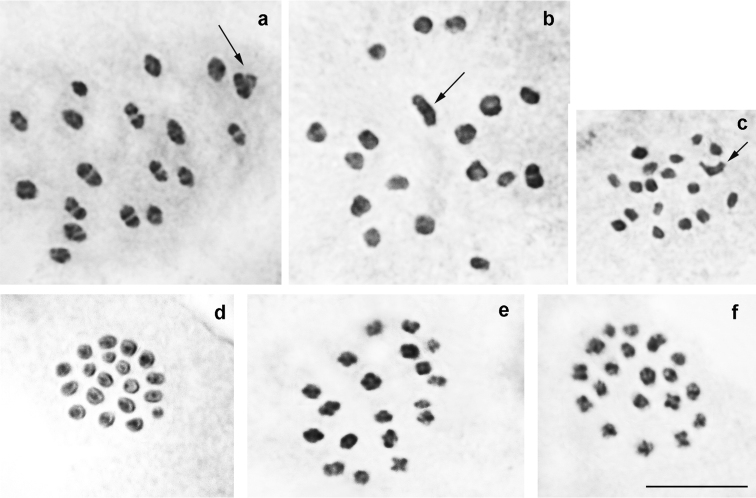
Karyotype of *P. damonides gilanensis* from its type-locality. Trivalents and triple chromatid are indicated by arrows. **a** sample J111, MI, n=18 **b** sample J111, MI, n=18 **c** sample J111, MII, n=18 **d** sample J112, MI, n=19 **e** sample J112, MI, n=19 f sample J112, MI, n=19. Bar = 10 μ.

**Figure 7.**
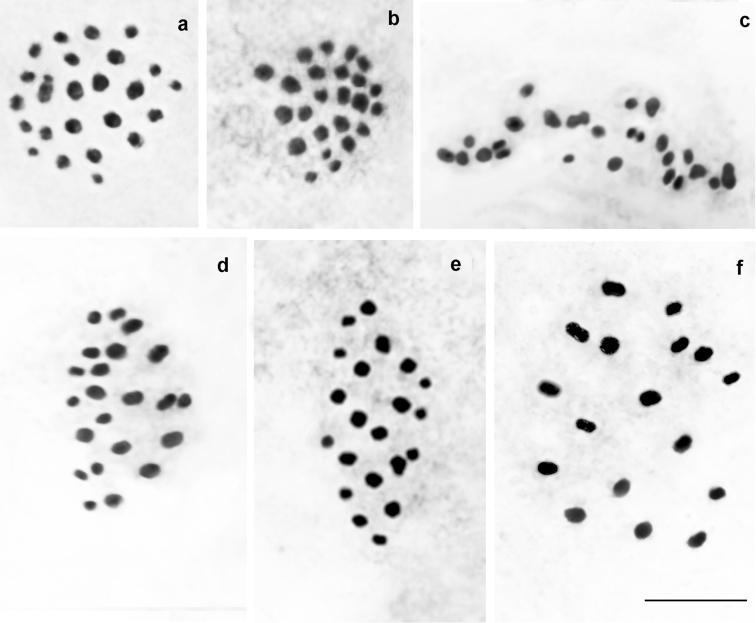
Karyotypes of *P. zarathustra*, *P. arasbarani*, *P. lukhtanovi* and *P. shamil*. **a** *P. zarathustra*, sample Z401, MI, n=24 **b** *P. arasbarani arasbarani*, sample N98, MI, n=24 **c** *P. arasbarani neglectus*, sample Q457, MI, n=ca25-26 **d** *P. lukhtanovi*, sample F875, MI, n=22 **e** *P. lukhtanovi*, sample H717, MI, n=21 **f** *P. shamil*, sample F958, MI, n=17. Bar = 10 μ.

157 specimens were karyotyped (Supplementary Table 1, Figs 2–7).

*P. ninae* (Fig. 2a–e)

At the MI/MII stages, the number of chromosome elements was found to vary from n=ca32 to n=34-36 in 21 studied specimens from different localities, with n=33 and n=34 as distinct modal numbers. All chromosome elements formed a gradient size row. The species seemed to be polymorphic for at least one chromosomal fusion/fission resulting in specimens possessing 33 bivalents (homozygotes for fused chromosomes) (Fig. 2b), 32 bivalents + 1 trivalents (heterozygotes for fusion/fission) (Fig. 2c, d) and 34 bivalents (homozygotes for unfused chromosomes) (Fig. 2a). Chromosomal rearrangements involved in formation of karyotypes with higher chromosome number (n=33-35 and n=34-36) remain still unknown.

*P. aserbeidschanus* (Fig. 2f–h)

At the MI/MII stages, the number of chromosome elements was found to vary from n=32 to n=37 in 17 studied specimens from different localities, with n=33 as a modal number. MI/MII metaphases consisted of elements of progressively decreasing size.

*P. australorossicus* sp. n. (Fig. 2i)

At the MI/MII stages, the haploid chromosome number n = 23 was found in 6 studied individuals. Elements were found to form a gradient size row in which the largest element was approximately 5 times larger than the smallest element. In two specimens, the diploid chromosome number was estimated as 2n = 46 in male atypical meiosis. In the sample DK-7-97 we counted approximately n = ca22 and in the sample from Chonkatau we counted approximately n = ca24 at the MI stage. The last two counts were done with an approximation due to the overlapping of some bivalents, therefore interpretation of these deviating numbers (a real variation or a mistake of counting) is difficult.

*P. damonides damonides* from Azerbaijan and Armenia (Fig. 3a–d)

At the MI/MII stages, the haploid chromosome number n = 18 was found in 10 studied individuals. Elements formed a gradient size row in which the largest element was approximately 2-2.5 times larger than the smallest element. In two specimens, the diploid chromosome number was determined as 2n = 36 in male atypical meiosis.

*P. damonides* from Iran (previously known as *P. elbursicus*) (Fig. 4a–h)

At the MI/MII stages, the haploid chromosome number n = 18 was found in 26 studied individuals. Elements constituted a gradient size row in which the largest element was approximately 2-2.5 times larger than the smallest element. In 7 specimens, the diploid chromosome number was determined as 2n = 36 in male atypical meiosis. Thus, the karyotype of these samples from Iran is indistinguishable from the karyotype of the samples of *P. damonides* from Azerbaijan and Armenia.

*P. damonides elbursicus* (Forster, 1956) (Fig. 5a–e)

At the MI/MII stages, the haploid chromosome number n = 17 was found in four studied individuals. Elements formed a gradient size row in which the largest element was approximately 2-2.5 times larger than the smallest element. In the sample VL311, the diploid chromosome number was determined as 2n = 34 in male atypical meiosis.

*P. damonides gilanensis* Eckweiler, 2002 (Fig. 6a–f)

At the MI/MII stages, the number of chromosome elements was found to vary from n=18 to n=19 in three studied specimens collected in the type-locality of this taxon. Elements formed a gradient size row in which the largest element was approximately 2-2.5 times larger than the smallest element. The population was found to be polymorphic for a chromosomal fusion/fission resulting in specimens possessing 18 bivalents (homozygotes for fused chromosomes), 17 bivalents + 1 trivalents (heterozygotes for fusion/fission) (Fig. 6a, b) and 19 bivalents (homozygotes for unfused chromosomes) (Fig. 6d–f). Interestingly, in the case of heterozygocity for fusion/fission, the same number of chromosome elements (n=18) was found at the MI and MII stages, and the trivalent chromosomes (triple chromatids) were observed at both MI and MII stages (Fig. 6a–c).

*P. zarathustra* (Fig. 7a)

At the MI/MII stages, the number of chromosome elements was found to vary from n=20-21 to n=24 in 6 studied specimens from different localities, with n=22 as a modal number. Elements formed a gradient size row in which the largest element was approximately 5 times larger than the smallest element. The species seemed to be polymorphic for several, still unrecognized chromosomal rearrangements resulting in chromosome number variation.

*P. arasbarani arasbarani* (Fig. 7b)

At the MI/MII stages, the number of chromosome elements was found to vary from n=23-24 to n=25 in 6 studied specimens, most likely due to polymorphism for a single chromosomal fusion/fission. Elements formed a gradient size row in which the largest element was approximately 5-6 times larger than the smallest element.

*P. arasbarani neglectus* Dantchenko, 2000 (Fig. 7c)

At the MI stage, the number of chromosome elements was determined to be n=25 in the sample B447. In the samples KA-95-99, 2001-Q456 and 2001-Q457 the number of elements was estimated with an approximation as n=24-25 and n=25-26 due to the overlapping of some bivalents. In the sample KA-98-99, the diploid chromosome number was estimated as 2n = ca 48. Elements formed a gradient size row in which the largest element was approximately 5-6 times larger than the smallest element.

*P. lukhtanovi* (Fig. 7d, e)

At the MI/MII stages, in 28 of 33 studied specimens the haploid chromosome number was determined as n=22. In one of these 28 specimens atypical meiosis displayed 2n=44. In 3 of 33 studied samples the haploid chromosome number was determined as n=21, and in two samples intraindividual variation in the number of elements was observed: n=21-22. We interpret this result as an evidence for polymorphism for a single fusion/fission resulting in in specimens possessing n=21 (Fig. 7e) and n=21-22 (homozygotes for fusion and heterozygotes for fusion/fission) and n=22 bivalents (homozygotes for the unfused chromosomes) (Fig. 7). Elements formed a gradient size row in which the largest element was approximately 3 times larger than the smallest element.

*P. shamil* (Fig. 7f)

At the MI/MII stages, in all 12 studied specimens the haploid chromosome number was determined as n=17. In three of these 12 specimens atypical meiosis displayed 2n=34. Elements formed a gradient size row in which the largest element was approximately 2 times larger than the smallest element.

### *COI* barcode analysis

**Figure 8.**
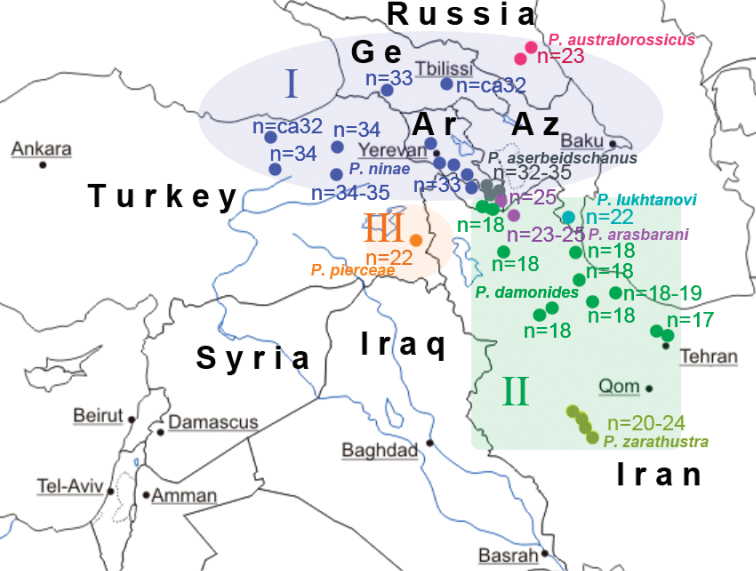
Distribution of *COI* haplogroups (I – III) and haploid chromosome numbers (n) in the *P. damonides* species complex.

The *COI* barcode analysis revealed three major, highly supported clusters within the studied samples (Fig. 1). The first cluster (haplogroup I) is represented by samples of *P. ninae*, *P. aserbeidschanus* and *P. australorossicus*. This cluster inhabits the northern part of the *P. damonides* complex distribution range: the Russian part of the eastern Caucasus (Daghestan), Georgia, Armenia (except its south-eastern part near Meghri), Nakhchivan in Azerbaijan (except Ordubad district) and north-eastern Turkey (Fig. 8).

Within its distribution range *P. ninae* demonstrates a diversity of *COI* haplotypes; however, no distinct intraspecific sublineages were discovered. *Polyommatus ninae firuze* (Carbonell, 1993) described from Turkey (Gümüşhane, Kelkit) shows no differentiation from topotypical populations from Armenia, and in our opinion should be considered no more than a synonym of *P. ninae*.

The specimens of *P. aserbeidschanus* collected in the type locality of this taxon “Armenia, mts. Zangezur, pag. Kadzharantz, pr. Mts. Kapudzhich” (Forster 1956) were found to share their *COI* haplotype with the samples of *P. ninae* from Armenia and Azerbaijan.

On the tree obtained, the samples of *P. australorossicus* were intermixed together with the samples of *P. ninae*. Although no shared haplotypes were found, the uncorrected *p*-distances between the *P. ninae* and *P. australorossicus* samples were quite low varying from 0.2 % to 1.4 %. Thus, both *P. ninae* and *P. australorossicus* appeared on the tree as genetically undifferentiated, non-monophyletic assemblages.

The second lineage (haplogroup II) is represented by samples of *P. damonides*, *P. elbursicus*, *P. elbursicus gilanensis*, *P. zarathustra*, *P. arasbarani* and *P. lukhtanovi*. This is the southern lineage of the *P. damonides* complex distributed in the south-eastern part of Nakhchivan (Ordubad district, Azerbaijan), south-eastern part of Armenia (Meghri district), south-eastern part of Azerbaijan (Talysh) and Iran (Fig. 8). On the tree obtained, the samples of this lineage were deeply intermixed, and all these taxa appeared as undifferentiated non-monophyletic assemblages.

The third lineage (haplogroup III) (south-western group) is represented by samples of *P. pierceae*. This lineage inhabits the south–eastern part of Turkey (Fig. 8).

## Discussion

### Rapid chromosomal evolution and possibility of chromosomal speciation

The *P. damonides* species complex demonstrates a high rate of karyotype evolution resulting in a great interspecific diversity of chromosome numbers (from n=17 to n 34-36) (Figs 2–7) despite the low (between the haplogroups I and II) or lack of (within the haplogroups I and II) (Fig. 1) *COI* differentiation. Fusions and fissions of chromosomes are the most probable rearrangements driving the chromosome number change in the *P. damonides* complex as well as in other butterfly species (Lukhtanov et al. 2011, Šíchová et al. 2015, 2016). In *P. ninae*, *P. damonides gilanensis*, *P. arasbarani* and *P. lukhtanovi* some of these fusions/fissions are found in heterozygous conditions resulting in trivalent formation at the MI stage.

There are two possible ways of the first meiotic division in a cell with the fusion/fission trivalent: (i) resulting in a daughter cell containing two smaller chromosomes and a daughter cell containing one larger chromosome, and (ii) resulting in two daughter cells, each containing an element consisting of three triple chromatids (Nokkala et al. 2006). In case of chiasmate meiosis, Nokkala et al. (2006) interpreted both ways as two different variants of conventional pre-reductional meiosis, whereas Banno et al. (1995) interpreted the second way as post-reductional meiosis. The latter is also known as “inverted meiosis” (see e.g. Heckman et al. 2014, Manicardi et al. 2015, Bogdanov 2016). Despite the difference in the interpretation (in fact despite the difference in the definition of conventional and inverted meiosis), both papers stress the significant distinction between the first and the second ways. In *P. damonides gilanensis* the trivalent-similar elements were found not only at the MI (Fig. 6a, b), but also at the MII stage (Fig. 6c), most likely due to the second way of the first meiotic division.

The fact that the discovered fusions/fissions can exist in populations in both homo- and heterozygous conditions indicates, most likely, that these rearrangements can pass through meiosis and are not strongly underdominant. Previously, the low or no underdominance of chromosomal fusions/fissions was demonstrated for butterflies of the genus *Leptidea* Billberg, 1820 (Lukhtanov et al. 2011, Šíchová et al. 2015, 2016). In the Agrodiaetus subgenus the low underdominance of chromosomal fusions/fissions was indirectly demonstrated through analysis of homoploid hybrid speciation in *P. karindus-P. morgani-P. peilei* species complex (Lukhtanov et al. 2015b). Particularly, the formation of the diploid hybrid species *P. peilei* Bethune-Baker, 1921 had to include a hybrid ancestor heterozygous for at least 41 single chromosome fusions/fissions, and this ancestor was at least partially fertile (Lukhtanov et al. 2015b). The low underdominance of the chromosomal fusions/fissions does not mean that these rearrangements are unimportant for the formation of reproductive isolation and speciation. The accumulation of multiple fusions/fissions can reduce gene flow between chromosomally divergent populations not only via (i) the hybrid-sterility mechanism (when chromosomal rearrangements reduce fertility of chromosomal heterozygotes), but also via (ii) the suppressed-recombination mechanism (even if chromosomal rearrangements are neutral and do not influence fertility of chromosomal heterozygotes) (Faria and Navarro 2010). Comparative phylogenetic analyses demonstrates that the second mechanism is more probable in *Agrodiaetus* (Vershinina and Lukhtanov 2017), and the gradual accumulation of chromosomal fusions-fissions can certainly drive speciation (Lukhtanov et al. 2005, Kandul et al. 2007).

Thus, the fixed differences in karyotype are not only (syn)apomorphic characters demonstrating that chromosomal races represents distinct phylogenetic lineages, i.e. species from the point of view of phylogenetic species concepts, but also indirect evidence for at least partial reproductive isolation.

### *COI* differentiation and taxonomy of the *P. damonides* complex

The studied complex demonstrates a high level of chromosomal differentiation between taxa and a relatively low level of differentiation with respect to the mitochondrial *COI* gene, with many distinct taxa intermixed on the *COI* tree obtained (Fig. 1). This result is quite expected taking into account the previous studies (Wiemers 2003, Kandul et al. 2004, Wiemers and Fiedler 2007, Lukhtanov and Shapoval 2017) that demonstrated low interspecific differenciation and even the presence of shared *COI* barcodes between several distinct species of *Agrodiaetus*. For chromosomally divergent species, such a situation can be explained by (i) a high rate of diversification in *Agrodiaetus* resulting in numerous young species sharing ancestral polymorphism for *COI* and/or (ii) occasional interspecific hybridization resulting in mitochondrial introgression (Kandul et al. 2004, Lukhtanov et al. 2005, Vishnevskaya et al. 2016). For example, both explanations can be applied to explain the molecular relationship between chromosomally divergent *P. ninae* and *P. australorossicus*, although the second explanation (mitochondrial introgression) seems to be much less probable given the current geographic isolation between them (Fig. 8).

In case of the pair *P. ninae* – *P. aserbeidschanus* which are indistinguishable in both molecular and chromosomal characters, we can also hypothesize that these two nominal taxa are conspecific. These two taxa have been long time considered as distinct species because of a wrong assumption about their karyotypic differentiation (Lukhtanov 1989). However, the analysis of karyotype of *P. ninae* from its type-locality (Armenia: vicinity of Azizbekov, now Vaik) (Lukhtanov 1989) and of *P. aserbeidschanus* from its type-locality (Armenia: vicinity of Kadzharan, now Kajaran) (this study) did not reveal any differences between them, and the molecular analysis demonstrated the identity of their *COI* barcodes (although nuclear genes have not been studied yet).

However, *P. ninae* and *P. aserbeidschanus* are not identical with respect to their morphology and ecological preferences. Male specimens of *P. aserbeidschanus* (mostly collected around the type locality in South Zangezur Range) have specific dark brown coloration on the wing underside, blue ground color with violet tint on the wing upperside and significantly smaller size compared with the males of *P. ninae*. *Polyommatus aserbeidschanus* is known only from the subalpine belt of the South Zangezur mountain area and connected trophically with the *Astragalus* species preliminary determined as *Astragalus prilipkoanus* (sectio *Incani*) (Fabaceae) (Dantchenko 2010). As it was shown previously, *Astragalus* species of the sectio *Incani* are also host plants for other taxa of the P. (A.) damonides species group (Dantchenko 2010). Typical males of *P. ninae* are larger in size and have blue (not violet) coloration of the upper surface of the wings. Typical *P. ninae* inhabits tragacanth communities in the Vayots Dzor mountain range and its hostplant is *Astragalus montis-aquilis* (sectio Incani) (Dantchenko 2010). Despite this morphological and ecological differentiation, *P. ninae* and *P. aserbeidschanus* can be theoretically interpreted as local intraspecific forms of the same species, and further studies are required to clarify this situation.

A similar case is found in the pair *P. zarathustra* – *P. arasbarani*. These two taxa are allopatric, and similar with respect to morphology, karyotypes and *COI* barcodes. However, they are differentiated with respect to ecological preferences: *P. zarathustra* is associated with dry areas in central Iran, whereas *P. arasbarani* is associated with meadow-like biotopes in subalpine zone of the north-west Iran. *Polyommatus arasbarani neglectus* is known only from low and middle altitude on southern slopes of the Meghri mountain range, it inhibits dry glades and clearance in an oak forest belt and trophically connected with astragalus species preliminary determined as *Astragalus fedorovi* (sectio *Incani*) (Fabaceae). This ecological differentiation does not allow synonymaizing these taxa, and further studies are required to clarify this situation, too.

*Lycaena Damone var. Damonides* Staudinger, 1899 is the oldest taxon described within the studied complex. Therefore analysis of its identity is of great importance for solving nomenclatural problems within the group. The taxon was described as a form of *Polyommatus damone* (hypothesis 1) and later considered as an entity close to *P. poseidon* (Forster 1961) (hypothesis 2), to *P. ninae* (Hesselbarth et al. 1995, p. 735, Eckweiler and Bozano 2016) (hypothesis 3) or to *P. elbursicus* (Lukhtanov in Hesselbarth et al. 1995, p. 735) (hypothesis 4) (see also Olivier et al. 1999, p. 16). Here we have analyzed the karyotype and *COI* barcodes of the samples from the type-locality (Ordubad in Nakhchivan, Azerbaijan) as well as the samples from the neighboring territory of Armenia (Meghri). Based on this material, we demonstrate that the hypothesis 4 is true. Thus, *P. damonides* appears as a taxon close and most likely conspecific with the taxon previously known as *P. elbursicus*. Therefore, we propose a taxonomic rearrangement of this group and suggest the following new combinations: *P. damonides elbursicus* Forster, 1956, comb. n. and *P. damonides gilanensis* Eckweiler, 2002, comb. n.

According to our observations *P. damonides damonides* inhabits tragacanth and *Paliurus* plant communities from 1000m. alt. (in Armenia) to 2100 m. alt. (in Nakhchivan, vicinity of Ordubad) and is trophically connected with *Astragalus ordubadensis* (sectio *Incani*) (Fabaceae) which is endemic of South Zangezur mountain range. It is also important to note that in Meghri-Ordubad region we have found sympatry/syntopy for the species pairs *P. arasbarani neglectus/P. damonidesdamonides*, *P. arasbarani neglectus/P. aserbeidschanus* and *P. damonides*/*P. aserbeidschanus*.

### New species description

#### Polommatus (Agrodiaetus) australorossicus sp. n.

Taxon classificationAnimaliaLepidopteraLycaenidae

http://zoobank.org/12D80F81-ECEB-4888-B148-A0D6AD3B8BC1

##### Holotype

**Figure 9.**
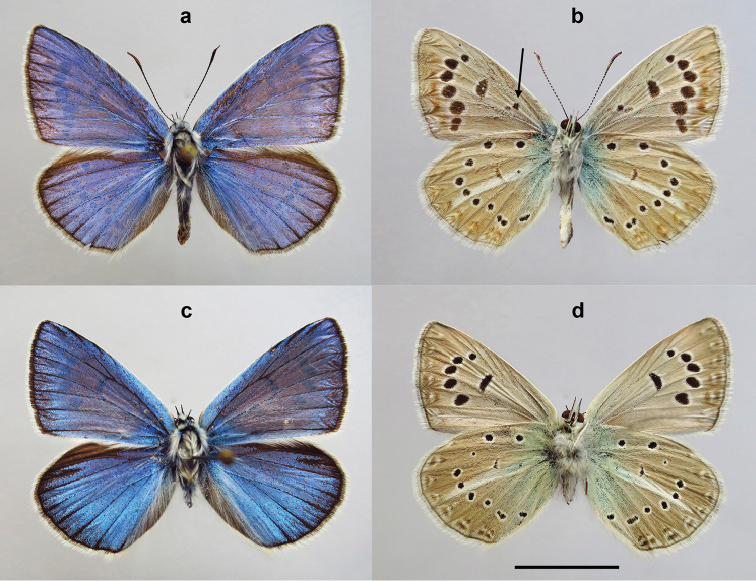
Specimens of Polyommatus (Agrodiaetus) australorossicus sp. n. and P. (A.) shamil. Both samples collected in Gunib (Russia, Caucasus, Daghestan, Gimrinsky Range, 1600-1800 m), 14 August 1997, by A. Dantchenko. **a, b** upperside (**a**) and underside (**b**) of the holotype of Polyommatus (Agrodiaetus) australorossicus sp. n. DK-27-97, n=23; arrow indicates basal black spot **c, d** upperside (**c**) and underside (**b**) of the paratype of Polyommatus (Agrodiaetus) shamil, CCDB-17947_B11, DK-97-18, n=17, 2n=34. Bar = 10 mm.

(Fig. 9a, b), male, BOLD process ID BPAL2013-13, field # CCDB-17947_B06, GenBank accession number MG243366; karyotype preparation DK-27-97, n=23; Russia, Caucasus, Daghestan, Gimrinsky Range, Gunib, 42.406274°N, 46.931548°E, 1680 m, 14 August 1997, A. Dantchenko leg., deposited in the Zoological Institute of the Russian Academy of Science (St. Petersburg).

##### *COI* barcode sequence of the holotype

(BOLD process ID BPAL2013-13; GenBank accession number MG243366).

ACATTATATTTTATTTTTGGAATTTGAGCAGGAATAGTAGGAACATCCNTAAGAATTTTAATTCGTATAGAATTGAGAA CTCCTGGATCCTTAATTGGAGATGATCAAATTTATAACACTATTGTTACAGCTCATGCATTTATTATAATTTTTTTTATA GTTATACCTATTATAATCGGAGGATTTGGTAACTGATTAGTTCCTTTAATATTAGGGGCACCTGATATAGCCTTTCCACG ACTAAATAATATAAGATTCTGATTATTACCGCCATCATTAATACTACTAATTTCCAGAAGAATTGTAGAAAATGGAGCAG GAACAGGATGAACAGTTTACCCCCCACTTTCATCTAATATTGCACATAGAGGATCATCTGTAGATTTAGCAATTTTCTCT CTTCATTTAGCAGGAATTTCTTCAATTTTAGGAGCAATTAATTTTATTACAACTATTATTAATATACGGGTAAATAATTT ATCTTTTGATCAAATATCATTATTTATTTGAGCAGTGGGAATTACAGCATTATTATTACTTTTATCTTTACCTGTATTAG CTGGAGCAATTACCATATTATTAACTGATCGAAATCTTAACACCTCATTCTTTGATCCAGCTGGTGGAGGAGATCCAATT TTATATCAACATTTA

##### Paratypes.

9 males. (1) BOLD process ID BPAL2011-13, field # CCDB-17947_B04; karyotype preparation DK-34-1-97; Russia, Caucasus, Daghestan, Gimrinsky Range, Gunib, 1800 m, 15 August 1997, A. Dantchenko leg. (2) BOLD process ID BPAL2012-13, field # CCDB-17947_B05; karyotype preparation DK-34-2-97, n=ca23; Russia, Caucasus, Daghestan, Gimrinsky Range, Gunib, 1800 m, 15 August 1997, A. Dantchenko leg. (3) BOLD process ID BPAL2014-13, field # CCDB-17947_B07; karyotype preparation DK-7-97, n=ca22; Russia, Caucasus, Daghestan, Gimrinsky Range, Gunib, 1800 m, 12 August 1997, A. Dantchenko leg. (4) karyotype preparation DK-23-97, n=23, 2n=46; Russia, Caucasus, Daghestan, Gimrinsky Range, Gunib, 1800 m, 15 August 1997, A. Dantchenko leg. (5) karyotype preparation DK-30-97, n=23; Russia, Caucasus, Daghestan, Gimrinsky Range, Gunib, 1800 m, 15 August 1997, A. Dantchenko leg. (6) karyotype preparation DK-23-97-3, n=23; Russia, Caucasus, Daghestan, Gimrinsky Range, Gunib, 1800 m, 14 August 1997, A. Dantchenko leg. (7) karyotype preparation DK-23-97-4, 2n=ca46; Russia, Caucasus, Daghestan, Gimrinsky Range, Gunib, 1800 m, 14 August 1997, A. Dantchenko leg. (8) karyotype preparation DK-27-97-2, n=23; Russia, Caucasus, Daghestan, Gimrinsky Range, Gunib, 1800 m, 14 August 1997, A. Dantchenko leg. (9) karyotype preparation n=?24; Russia, Caucasus, Daghestan, Chonkatau, V. Tikhonov leg. All paratypes are deposited in the Zoological Institute of the Russian Academy of Science (St. Petersburg).

##### Additional samples

(no DNA, no karyotype). 10 males: Russia, Caucasus, Daghestan, Gimrinsky Range, Gunib, 1450–1950 m, 11–16 August 1997, A. Dantchenko leg.

***Males.*** Forewing length 16.5–18.5 mm.

Upperside: Ground colour bright glossy violet blue with narrow black marginal line, marginal part of forewings and hindwings slightly dusted with black scales, discal strokes absent, veins darkened distally, costal area of the forewings white, basal part of fringe dark grey on forewings, light grey on hindwings, distal part white.

Underside: Forewing ground colour grey, submarginal row blurred, but clear visible; discoidal strokes black, bordered with white; postdiscal rows of black spots bordered with white, 80% males have basal black spots; hindwing ground colour grey with ocherous tint, basal area with strong greenish suffusion; discal stroke less prominent than on forewings; postdiscal row of black spots bordered with white, submarginal and antemarginal marking not strong but clear visible; submarginal row bordered distally with reddish brackets, more pronounced to anal end of row; white streak sharp, equal in width; basal half of fringes pale grayish on fore- and hindwings, distal part white.

***Females*** remains unknown.

***Genitalia.*** The male genitalia have a structure typical for other species of the subgenus Agrodiaetus (Coutsis 1986).

##### Habitat and biology.

Stony steppe and dry meadows from 1500 up to 2000 m a.s.l. Flight period: mid-July to end of August, in a single generation. The new species flights syntopically and synchronously with *P. shamil* but on average about one decade earlier. Host plant is preliminary determined as *Astragalus buschiorum* (Fabaceae). Hibernation as first instar larvae.

##### Diagnosis.

**Figure 10.**
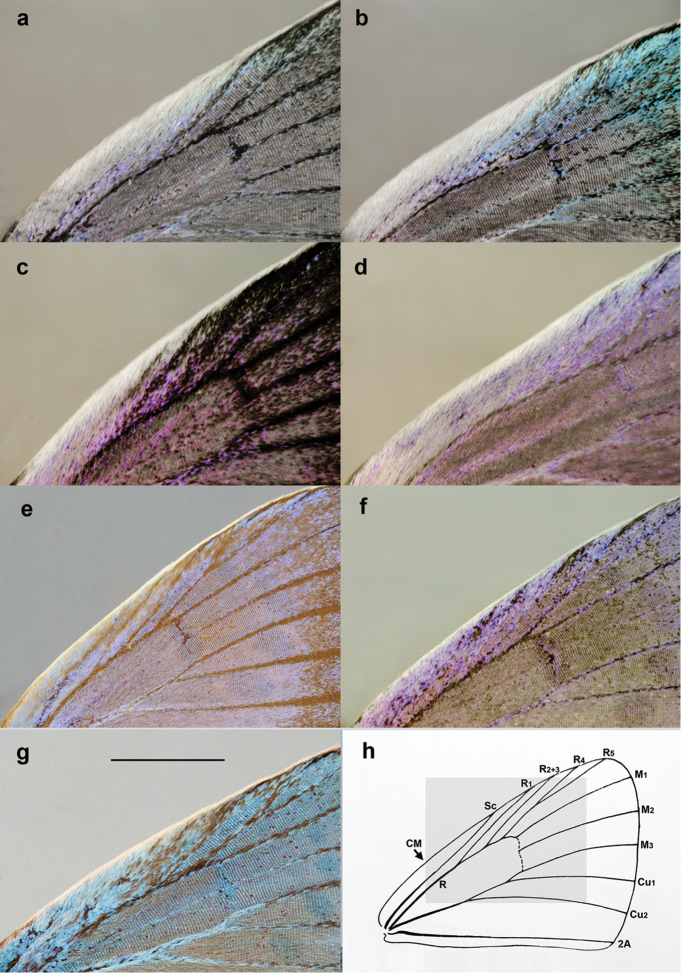
Pubescence of the anterior part of the forewing upper surface. **a, b, c, d** the anterior part of the forewing upper surface possesses a strong white pubescence in the area bordered by the costal margin (CM) and the veins R and R_2+3_, i.e. this area is densely covered with relatively long white hairs (**a** *P. aserbeidschanus* **b** *P. ninae*, **c** *P. arasbarani neglectus* **d** *P. australorossicus*) **e, f** the white pubescence of the anterior part of the forewing upper surface is strongly reduced and limited to the only costal margin, the white hairs are short (**e** *P. ciscaucasicus* **f** *P. shamil*) **g** the white pubescence of the anterior part of the forewing upper surface is reduced, not dense (*P. damonides*) **h** schematic picture showing the venation of the forewing in *Polyommatus* and the photographed area (shaded). Bar = 3 mm.

Phenotypically P. (A.) australorossicus sp. n. is practically indistinguishable from allopatric closely related *P. ninae*, *P. aserbeidschanus* and *P. lukhtanovi* but the ground colour of the underside of the hindwings is grey in the new species, with ocherous tint, not light or dark brown. The new species differs from sympatric (syntopic and synchronous) *P. shamil* (Fig. 9c, d) by specific structure of costal area of the forewings in males (Fig. 10). The submarginal row of spots on the forewing underside is more blurred (Fig. 9b), not sharp and clear visible as in *P. shamil* (Fig. 9d). Additionally, basal black spots are usually present on the underside of the forewings in P. (A.) australorossicus (Fig. 9b); however, this character is not constant.

Genetically *P. australorossicus* and *P. shamil* are not close. They belong to two different species groups within the subgenus Agrodiaetus: to *P. carmon* group (*P. australorossicus*) and to *P. cyaneus* group (*P. shamil*).

The new species differs drastically from the genetically most closely related *P. ninae* and *P. aserbeidschanus* by its karyotype (by at least 9 fixed chromosomal fusions/fissions).

The new species is similar (but not identical) to *P. lukhtanovi* (n=21-22) and *P. pierceae* (n=22) with respect to the chromosome number. However, it differs from these species by *COI* barcodes and represents a different lineage of evolution within the *P. damonide* complex.

##### Etymology.

The name *australorossicus* is an adjective of the masculine gender. This species name originates from the Latin words “australis” (south) and “rossicus” (Russian).

### Taxonomic conclusion

We propose the following taxonomic arrangement of the *P. damonides* species complex (chromosome numbers are in parentheses):

**i. Polyommatus (Agrodiaetus) ninae lineage**

P. (A.) ninae (Forster, 1956) (*Agrodiaetus transcaspica ninae* Forster, 1956; =*Agrodiaetus ninae firuze* Carbonell, 1993) (n=33-35)

P. (A.) aserbeidschanus (Forster, 1956) (*Agrodiaetus transcaspica aserbeidschana* Forster, 1956) (n=32-37)

P. (A.) australorossicus, sp. n. (n=23)

**ii. Polyommatus (Agrodiaetus) damonides lineage**

P. (A.). *damonides* (Staudinger,1899)

P. (A.) damonides damonides (Staudinger, 1899) (*Lycaena Damone var. Damonides* Staudinger, 1899) (n=18)

P. (A.) damonides elbursicus (Forster, 1956), comb. n. (*Agrodiaetus transcaspica elbursica* Forster, 1956) (n=17)

P. (A.) damonides gilanensis Eckweiler, 2002, comb. n. (Polyommatus (Agrodiaetus) elbursicus gilanensis Eckweiler, 2002) (n=18-19)

P. (A.) lukhtanovi (Dantchenko, 2005) (*Agrodiaetus lukhtanovi* Dantchenko, 2005) (n=21-22)

P. (A.) zarathustra Eckweiler, 1997 (Polyommatus (Agrodiaetus) zarathustra Eckweiler, 1997) (n=20-24)

P. (A.) arasbarani (Carbonel & Naderi, 2000)

P. (A.) arasbarani arasbarani Carbonel & Naderi, 2000 (*Agrodiaetus arasbarani* Carbonel & Naderi, 2000) (n=24-25)

P. (A.) arasbarani neglectus Dantchenko, 2000 (Polyommatus (Agrodiaetus) zarathustra neglectus Dantchenko, 2000; = Polyommatus (Agrodiaetus) arasbarani ihmal Koçak & Kemal, 2008) (n=24-26)

**iii. Polyommatus (Agrodiaetus) pierceae lineage**

P. (A.) pierceae (Lukhtanov & Dantchenko, 2002) (*Agrodiaetus pierceae* Lukhtanov & Dantchenko, 2002) (n=22)

**Comment**. The name Polyommatus (Agrodiaetus) arasbarani ihmal was suggested by Koçak and Kemal (2008) to replace Polyommatus (Agrodiaetus) zarathustra neglectus Dantchenko, 2000. Koçak and Kemal (2008) assumed that Polyommatus (Agrodiaetus) zarathustra neglectus Dantchenko, 2000 was a junior homonym of *Polyommatus neglectus* Stradomsky & Arzanov [2000], a species close to *Polyommatus icarus* (Rottemburg, 1775) described by Stradomsky and Arzanov in the second issue of the volume 7 of Izvestiya Kharkovskogo Entomologicheskogo Obschestva (p. 19) (Stradomsky and Arzanov [2000]). This issue is dated by the year 2000; however, the real date is not clear. As is written on the page 172 the issue was signed for printing on December 21, 1999, but the day when it was really printed and became accessible is unknown. This issue appeared in the library of the Zoological Institute of the Russsian Academy of Science on July 18, 2000. Thus we assume that it was published between December 21, 1999 and July 18, 2000.

The volume 48 of Neue Entomologische Nachrichten with description of Polyommatus (Agrodiaetus) zarathustra neglectus Dantchenko, 2000 was published and distributed in May 2000. Additional studies are required to clarify what taxon (described by Dantchenko or described by Stradomsky and Arzanov) was published first. Until this situation is resolved in a future revision, we see no other way than to use P. (A.) arasbarani neglectus Dantchenko, 2000 as a valid name.

## Supplementary Material

XML Treatment for Polommatus (Agrodiaetus) australorossicus

## Supplementary materials

Supplementary material 1 Table S1 Link: https://doi.org/10.3897/CompCytogen.v11i4.20072.suppl1 Data type: PDF table. Explanation note: Chromosome numbers of the studied Polyommatus (Agrodiaetus) samples with their IDs, localities, dates and collectors. This dataset is made available under the Open Database License (http://opendatacommons.org/licenses/odbl/1.0/). The Open Database License (ODbL) is a license agreement intended to allow users to freely share, modify, and use this Dataset while maintaining this same freedom for others, provided that the original source and author(s) are credited.Vladimir A. Lukhtanov, Alexander V. Dantchenko
