# Supplementary material for: A new butterfly species from south Russia revealed through chromosomal and molecular analysis of the Polyommatus (Agrodiaetus) damonides complex (Lepidoptera, Lycaenidae)
Source: Comp Cytogenet. 2017 Nov 24;11(4):769–95. doi: 10.3897/CompCytogen.v11i4.20072 (PMC5740405; doi:10.3897/CompCytogen.v11i4.20072)
Supplement: Supplementary material 1 — Table S1 [file comparative_cytogenetics-11-769-s001.pdf]

Table I.

Chromosome numbers of the studied samples with their ID, localities dates and collectors

1 AD = Alexander Danchenko, VL = Vladimir Lukhtanov

Taxon	Sample ID number	COI ID number	Chromosome number	Country	Locality	Date	Collector(s)
P. arasbarani arasbarani	N078		n=ca23-25	Iran	E Azerbaijan Prov., W Keleybar, Mekidu, 1900–2000m	29 July 2005	VL & AD
P. arasbarani arasbarani	N087		n=25	Iran	E Azerbaijan Prov., W Keleybar, Mekidu, 1900–2000m	29 July 2005	VL & AD
P. arasbarani arasbarani	N090		n=24	Iran	E Azerbaijan Prov., W Keleybar, Makhmutobad, 2100m	29 July 2005	VL & AD
P. arasbarani arasbarani	N093		n=25	Iran	E Azerbaijan Prov., W Keleybar, Makhmutobad, 2100m	29 July 2005	VL & AD
P. arasbarani arasbarani	N097		n=24	Iran	E Azerbaijan Prov., W Keleybar, Makhmutobad, 2100m	29 July 2005	VL & AD
P. arasbarani arasbarani	N098		n=24	Iran	E Azerbaijan Prov., W Keleybar, Makhmutobad, 2100m	29 July 2005	VL & AD
P. arasbarani neglectus	KA-98-99		2n=ca48	Armenia	Meghri, Schwanidzor	24 July 1999	AD
P. arasbarani neglectus	KA-95-99		n=24-25	Armenia	Meghri, Schwanidzor	25 July 1999	AD
P. arasbarani neglectus	2001-Q456		n=25-26	Armenia	Gyumorats	2001	AD
P. arasbarani neglectus	2001-Q457		n=25-26	Armenia	Gyumorats	2001	AD
P. arasbarani neglectus	B447		n=25	Armenia	Meghri, Shvanidzor	1 July 2001	AD
P. aserbeidschanus	Nr 17-2001		n=32	Armenia	Kadzharan, Katnarat	4 August 2001	AD
P. aserbeidschanus	Q470-472		n=32-33	Armenia	Kadzharan, Katnarat	4 August 2001	AD
P. aserbeidschanus	B273, ПХ-18		n=caca30	Armenia	Kadzharan, Pkhrut vill., 1900 m	2 August 2002	AD
P. aserbeidschanus	B274, ПХ-26		2n=ca68-70	Armenia	Kadzharan, Pkhrut vill., 1900 m	2 August 2002	AD
P. aserbeidschanus	B276, ПХ-30		n=34	Armenia	Kadzharan, Pkhrut vill., 1900 m	2 August 2002	AD
P. aserbeidschanus	05A387		n=33	Armenia	Kadzharan, Pkhrut vill.	15 August 2005	AD
P. aserbeidschanus	05A388		n=33	Armenia	Kadzharan, Pkhrut vill.	15 August 2005	AD
P. aserbeidschanus	J241		n=33	Armenia	Sevan	9 August 2005	AD
P. aserbeidschanus	J242		n=ca33	Armenia	Sevan	9 August 2005	AD
P. aserbeidschanus	J244		n=33-35	Armenia	Sevan	9 August 2005	AD
P. aserbeidschanus	05A406		n=32	Armenia	Kirs	17 August 2005	AD
P. aserbeidschanus	05A408		n=33	Armenia	Kirs	17 August 2005	AD
P. aserbeidschanus	J240		n=33, 2n=68	Armenia	Sevan	9 August 2005	AD
P. aserbeidschanus	05A405		n=32	Armenia	Kirs	17 August 2005	AD
P. aserbeidschanus	160K16A		n=37	Armenia	Syunik, Geghi vill., 39.234981°N, 46.142446°E, 1640m	12 August 2016	AD
P. aserbeidschanus	161K16A		n=33	Armenia	Syunik, Geghi vill., 1640m	12 August 2016	AD
P. aserbeidschanus	167K16A		n=34	Armenia	Syunik, Geghi vill., 1640m	12 August 2016	AD

Taxon	Sample ID number	COI ID number	Chromosome number	Country	Locality	Date	Collector(s)
P. australorossicus	DK-7-97	CCDB-17947_B07	n=ca22	Russia	Daghestan, Gunib	12 August 1997	AD
P. australorossicus	DK-23-97		n=23	Russia	Daghestan, Gunib	14 August 1997	AD
P. australorossicus	DK-30-97-1		n=23	Russia	Daghestan, Gunib	15 August 1997	AD
P. australorossicus	DK-30-97-3		n=23, 2n=46	Russia	Daghestan, Gunib	15 August 1997	AD
P. australorossicus	DK-30-97-4		2n=ca46	Russia	Daghestan, Gunib	15 August 1997	AD
P. australorossicus	DK-34-97-2	CCDB-17947_B05	n=ca23	Russia	Daghestan, Gunib	15 August 1997	AD
P. australorossicus	DK-27-97-1	CCDB-17947_B06 Holotype	n=23	Russia	Daghestan, Gunib	14 August 1997	AD
P. australorossicus	DK-27-97-2		n=23	Russia	Daghestan, Gunib	14 August 1997	AD
P. australorossicus	Chonkatau		n=?24	Russia	Daghestan, Chonkatau		V. Tikhonov
P. damonides	2014VL01	CCDB-17968_A10	n=18	Azerbaijan	Nakhchivan, Ordubad, Nyus-Nyus	12 July 2014	VL
P. damonides	2014VL02		n=18	Azerbaijan	Nakhchivan, Ordubad, Nyus-Nyus	12 July 2014	VL
P. damonides	2014VL03		2n=36	Azerbaijan	Nakhchivan, Ordubad, Nyus-Nyus	12 July 2014	VL
P. damonides	2014VL04		2n=36	Azerbaijan	Nakhchivan, Ordubad, Nyus-Nyus	12 July 2014	VL
P. damonides	2014VL11	CCDB-17968_A11	n=18	Azerbaijan	Nakhchivan, Ordubad, Nyus-Nyus	12 July 2014	VL
P. damonides	2014VL13	CCDB-17968_A12	n=18	Azerbaijan	Nakhchivan, Ordubad, Nyus-Nyus	12 July 2014	VL
P. damonides	2014VL25		n=18	Azerbaijan	Nakhchivan, Ordubad, Nyus-Nyus	12 July 2014	VL
P. damonides	002A14K	CCDB-17968_A07	n=18	Armenia	Meghri	June 2014	AD
P. damonides	004A14K		n=18	Armenia	Meghri	June 2014	AD
P. damonides	005A14K	CCDB-17968_A08	n=18	Armenia	Meghri	June 2014	AD
P. damonides	006A14K		n=18	Armenia	Meghri	June 2014	AD
P. damonides	007A14K		n=18	Armenia	Meghri	June 2014	AD
A. elbursicus	VL302		n=17	Iran	Kendevan-1	1 August 2002	VL & AD
A. elbursicus	VL303		n=17	Iran	Kendevan-1	1 August 2002	VL & AD

Taxon	Sample ID number	COI ID number	Chromosome number	Country	Locality	Date	Collector(s)
A. elbursicus	VL311	AY953999	n=17, 2n=34	Iran	Kendevan-1	1 August 2002	VL & AD
A. elbursicus	M804		n=17	Iran	Tehran Prov., Dizin, 2700–2800 m	4 August 2005	VL & AD
A. elbursicus	VL451		n=18	Iran	Masuleh	3 August 2002	VL & AD
A. elbursicus	VL453		n=18	Iran	Masuleh	3 August 2002	VL & AD
A. elbursicus	VL454		n=18	Iran	Masuleh	3 August 2002	VL & AD
A. elbursicus	VL456		n=18	Iran	Masuleh	3 August 2002	VL & AD
A. elbursicus	VL486		n=18	Iran	Masuleh	3 August 2002	VL & AD
A. elbursicus	E155		n=18, 2n=36	Iran	Zanjan (W part), 10 km W Dandy, 1900m, 2004.VII.14	14 July 2004	VL & AD
A. elbursicus	E157		n=18	Iran	Zanjan (W part), 10 km W Dandy, 1900m, 2004.VII.14	14 July 2004	VL & AD
A. elbursicus	E164		n=18	Iran	Zanjan (W part), 10 km W Dandy, 1900m, 2004.VII.14	14 July 2004	VL & AD
A. elbursicus	E231		n=18, 2n=36	Iran	10 km N Enar, N slopes of Savalan, 1700m	18 July 2004	VL & AD
A. elbursicus	E193		n=18	Iran	Zanjan (W part), 10 km W Dandy, 1900m, 2004.VII.14	14 July 2004	VL & AD
A. elbursicus	E233		n=18	Iran	10 km N Enar, N slopes of Savalan, 1700m	18 July 2004	VL & AD
A. elbursicus	E234		n=18, 2n=36	Iran	10 km N Enar, N slopes of Savalan, 1700m	18 July 2004	VL & AD
A. elbursicus	E237		n=18, 2n=36	Iran	10 km N Enar, N slopes of Savalan, 1700m	18 July 2004	VL & AD
A. elbursicus	E238		n=18	Iran	10 km N Enar, N slopes of Savalan, 1700m	18 July 2004	VL & AD
A. elbursicus	E246		n=18	Iran	10 km N Enar, N slopes of Savalan, 1700m	18 July 2004	VL & AD
A. elbursicus	E460		n=18	Iran	Azerbaijan-e-Gharbi, Takab, 10 km E Takht-e-Suleyman, 2300–2500 m	31 July 2004	VL & AD
A. elbursicus	E459		n=18	Iran	Azerbaijan-e-Gharbi, Takab, 10 km E Takht-e-Suleyman, 2300–2500 m	31 July 2004	VL & AD
A. elbursicus	E469		n=18	Iran	Azerbaijan-e-Gharbi, Takab, 10 km E Takht-e-Suleyman, 2300–2500 m	31 July 2004	VL & AD
A. elbursicus	E470		n=18, 2n=36	Iran	Azerbaijan-e-Gharbi, Takab, 10 km E Takht-e-Suleyman, 2300–2500 m	31 July 2004	VL & AD
A. elbursicus	E471		n=18	Iran	Azerbaijan-e-Gharbi, Takab, 10 km E Takht-e-Suleyman, 2300–2500 m	31 July 2004	VL & AD
A. elbursicus	J573		n=18, 2n=36	Iran	30 km NE Zanjan, 5 km to East from the 1st pass	25 July 2005	VL & AD
A. elbursicus	J574		n=18	Iran	30 km NE Zanjan, 5 km to East from the 1st pass	25 July 2005	VL & AD
A. elbursicus	J608		n=18	Iran	E Azerbaijan Prov., Sahand Mt. S Tabriz, 2000 m	26 July 2005	VL & AD
A. elbursicus	J609		n=18	Iran	E Azerbaijan Prov., Sahand Mt. S Tabriz, 2000 m	26 July 2005	VL & AD

Taxon	Sample ID number	COI ID number	Chromosome number	Country	Locality	Date	Collector(s)
A. elbursicus	J614		n=18	Iran	E Azerbaijan Prov., Sahand Mt. S Tabriz, 2000 m	26 July 2005	VL & AD
A. elbursicus	J615		n=18, 2n=36	Iran	E Azerbaijan Prov., Sahand Mt. S Tabriz, 2000 m	26 July 2005	VL & AD
A. elbursicus gilanensis	J111		n=18	Iran	Gilan, Rudbar, Kelishom, 2000–2100 m	3 August 2004	VL & AD
A. elbursicus gilanensis	J109	EF104637	n=18	Iran	Gilan, Rudbar, 10 km W Baresar, 1300 m	3 August 2004	VL & AD
A. elbursicus gilanensis	J112		n=19	Iran	Gilan, Rudbar, Jirandeh, 1500–1600 m	3 August 2004	VL & AD
A. lukhtanovi	95A09		n=22	Azerbaijan	Talysh, Zuvand	20 June 1995	AD
A. lukhtanovi	95A10		n=22	Azerbaijan	Talysh, Zuvand	20 June 1995	AD
A. lukhtanovi	95ДБ5		n=21	Azerbaijan	Talysh, Zuvand	20 June 1995	AD
A. lukhtanovi	F869	AY954021	n=22	Azerbaijan	Talysh, Mistan	30 July 2003	AD & VL
A. lukhtanovi	H702		n=22	Azerbaijan	Talysh, Mistan	4 August 2003	AD
A. lukhtanovi	H708		n=ca22	Azerbaijan	Talysh, Mistan	4 August 2003	AD
A. lukhtanovi	F870		n=22	Azerbaijan	Talysh, Mistan	30 July 2003	AD & VL
A. lukhtanovi	F873		n=22	Azerbaijan	Talysh, Mistan	30 July 2003	AD & VL
A. lukhtanovi	F882		n=22	Azerbaijan	Talysh, Mistan	30 July 2003	AD & VL
A. lukhtanovi	F884	Holotype	n=22	Azerbaijan	Talysh, Mistan	30 July 2003	AD & VL
A. lukhtanovi	F885		n=22	Azerbaijan	Talysh, Mistan	30 July 2003	AD & VL
A. lukhtanovi	F886		n=22	Azerbaijan	Talysh, Mistan	30 July 2003	AD & VL
A. lukhtanovi	F888		n=22	Azerbaijan	Talysh, Mistan	30 July 2003	AD & VL
A. lukhtanovi	H705		n=22	Azerbaijan	Talysh, Mistan	4 August 2003	AD
A. lukhtanovi	H709		n=22	Azerbaijan	Talysh, Mistan	4 August 2003	AD
A. lukhtanovi	H711		n=21	Azerbaijan	Talysh, Mistan	4 August 2003	AD
A. lukhtanovi	H717		n=ca21-22	Azerbaijan	Talysh, Mistan	4 August 2003	AD
A. lukhtanovi	A. sp. 2-N1		n=22	Azerbaijan	Talysh, Mistan	5 August 2003	AD
A. lukhtanovi	A. sp. 2-N2		n=22	Azerbaijan	Talysh, Mistan	5 August 2003	AD
A. lukhtanovi	A. sp. 2-N11		n=21	Azerbaijan	Talysh, Mistan	5 August 2003	AD
A. lukhtanovi	A. sp. 2-N12		n=22	Azerbaijan	Talysh, Mistan	5 August 2003	AD
A. lukhtanovi	A. sp. 6R		n=22	Azerbaijan	Talysh, Mistan	7 August 2003	AD
A. lukhtanovi	A. sp. 7R		n=22	Azerbaijan	Talysh, Mistan	7 August 2003	AD
A. lukhtanovi	A. sp. 1 -N15		n=ca21-22	Azerbaijan	Talysh, Mistan	2 August 2003	AD
A. lukhtanovi	A. sp. 1 -N1		n=22	Azerbaijan	Talysh, Mistan	30 July 2003	AD

Taxon	Sample ID number	COI ID number	Chromosome number	Country	Locality	Date	Collector(s)
A. lukhtanovi	A. sp. 1 -N7		n=22, 2n=44	Azerbaijan	Talysh, Mistan	30 July 2003	AD
A. lukhtanovi	A. sp. 1 -N13		n=22	Azerbaijan	Talysh, Mistan	30 July 2003	AD
A. lukhtanovi	H719		n=22	Azerbaijan	Talysh, Mistan	30 July 2003	AD
A. lukhtanovi	1984-237		n=22	Azerbaijan	Talysh, Galobin	22-23 July 1984	VL
A. lukhtanovi	1984-317		n=22	Azerbaijan	Talysh, Galobin	22-23 July 1984	VL
A. lukhtanovi	1984-320		n=22	Azerbaijan	Talysh, Galobin	22-23 July 1984	VL
A. lukhtanovi	1984-321		n=22	Azerbaijan	Talysh, Galobin	22-23 July 1984	VL
A. lukhtanovi	1984-322		n=22	Azerbaijan	Talysh, Galobin	22-23 July 1984	VL
A. ninae	1984-206		n=ca29-34	Armenia	Daralagez mts, Azizbekov distr., Arpa-Chai	13 July 1984	VL
A. ninae	1984-212		n=34	Armenia	Daralagez mts, Azizbekov distr., Arpa-Chai	13 July 1984	VL
A. ninae	KL-36-96		n=34-36	Armenia	Aragaz, Ambert	22 July 1996	AD
A. ninae	KL-59-97		n=33-35	Armenia	Gnyshik	18-20 July 1997	AD
A. ninae	KL-72-97		n=34	Armenia	Gnyshik	18-20 July 1997	AD
A. ninae	KL-51-97		n=33-34	Armenia	Gnyshik	16 July 1997	AD
A. ninae	2001-191, L281		n=34	Turkey	Tercan	17 July 2001	AD & VL
A. ninae	2001-566, L413		n=34	Turkey	Prov. Erzurum, 8 km W Kayabasi	28 July 2001	AD & VL
A. ninae	2001-640, L434		n=34	Turkey	Tortum	30 July 2001	AD & VL
A. ninae	2001-566, L413		n=35	Turkey	Prov. Erzurum, 8 km W Kayabasi	28 July 2001	AD & VL
A. ninae	2001-084, L107		n=ca32	Turkey	Torul, 2nd place	13 July 2001	AD & VL
A. ninae	ch.6		n=ca32	Georgia	Tbilisi, Tcherepashje lake		AD
A. ninae	2014VL027		n=33	Azerbaijan	Nakhchivan, Badamly	14 July 2014	VL
A. ninae	2014VL032	CCDB-17968_H07	n=33	Azerbaijan	Nakhchivan, Badamly	14 July 2014	VL
A. ninae	2014VL033		n=33	Azerbaijan	Nakhchivan, Badamly	14 July 2014	VL
P. ninae	2014VL034		n=33	Azerbaijan	Nakhchivan, Badamly	14 July 2014	VL
P. ninae	2014VL038		n=33	Azerbaijan	Nakhchivan, Badamly	14 July 2014	VL
P. ninae	2014VL039	CCDB-17968_H08	n=33	Azerbaijan	Nakhchivan, Badamly	14 July 2014	VL
P. ninae	2014VL060	CCDB-17968_B05	n=33	Georgia	Akhalcikhe	18 July 2014	VL

Taxon	Sample ID number	COI ID number	Chromosome number	Country	Locality	Date	Collector(s)
A. ninae firuzae	2001-154, L169	AY496768	n=34	Turkey	Dilekyulu	16 July 2001	AD & VL
A. ninae firuzae	2001-157, L172		n=34	Turkey	Dilekyulu	16 July 2001	AD & VL
P. pierceae	92113		n=22	Turkey	Guzeldere Gecidi, SW side 2650–2850 m	4-5 August 1992	W. De Prins
P. pierceae	92114		n=22	Turkey	Guzeldere gecidi, SW side, 2650–2850 m	4-5 August 1992	W. De Prins
P. pierceae	2001-439,L365	AY496773 Holotype	n=22	Turkey	Guzuldere Gecidi	24 July 2001	AD & VL
P. shamil	DK-44-97	CCDB-17947_B08	n=17	Russia	Daghestan, Gunib	15 August 1997	AD
P. shamil	DK-55-97	CCDB-17947_B12	n=17	Russia	Daghestan, Gunib	16 August 1997	AD
P. shamil	DK-46-97	CCDB-17947_B09	n=17	Russia	Daghestan, Gunib	15 August 1997	AD
P. shamil	DK-97-18	CCDB-17947_B11	n=17, 2n=34	Russia	Daghestan, Gunib	14 August 1997	AD
P. shamil	DK-19-97		n=17, 2n=34	Russia	Daghestan, Gunib	14 August 1997	AD
P. shamil	DK-18-97		n=17	Russia	Daghestan, Gunib	15 August 1997	AD
P. shamil	DK-35-97		n=17, 2n=34	Russia	Daghestan, Gunib	15 August 1997	AD
P. shamil	F958		n=17	Azerbaijan	Altyagach	2 August 2003	VL
P. shamil	H613		n=17	Azerbaijan	Altyagach	4 August 2003	VL
P. shamil	Chonkatau-01		n=17	Russia	Daghestan, Chonkatau		V. Tikhonov
P. shamil	Chonkatau-02		n=17	Russia	Daghestan, Chonkatau		V. Tikhonov
P. shamil	Chonkatau-02		n=ca17	Russia	Daghestan, Chonkatau		V. Tikhonov
P. zarathustra	SKALA-2001-54		n=22	Iran	Kuh-e-Garin	3 August 2001	P. Skala
P. zarathustra	WE02531	AY557141	n=ca22	Iran	Lorestan, Dorud	25 July 2002	W. Eckweiler
P. zarathustra	N573	AY953994	n=20-21	Iran	Razan Pass, 33–33N; 48–50E	13 July 2002	VL
P. zarathustra	WE02533		n=ca22	Iran	Iran, 30 km W Dorud	25 July 2002	W. Eckweiler
P. zarathustra	N572		n=20-21	Iran	Razan Pass, 33–33N; 48–50E	13 July 2002	VL
P. zarathustra	Z401		n=24	Iran	Lorestan, W of Borujerd, Kuh-e-Garin, Vanai	21 July 2007	VL & N. Shapoval
